# Effect of Preorthodontic Trainer in Mixed Dentition

**DOI:** 10.1155/2013/717435

**Published:** 2013-12-04

**Authors:** Pallavi Pujar, Suryakanth M. Pai

**Affiliations:** ^1^Department of Pedodontics, Maratha Mandal's Dental College, Belgaum 590010, Karnataka, India; ^2^Department of Pedodontics, College of Dental Sciences, Davangere 577004, India

## Abstract

*Background*. This paper reports a case with end-on-molar relation with maxillary anterior teeth proclination, lower anterior teeth crowding, and deep bite. The case was successfully treated in a short span. Clinically significant improvement in the dimensions of the maxillary and mandibular dental arch forms and sufficient space for the eruption of permanent teeth was observed. *Conclusion*. Therefore, it can be concluded that *preorthodontic trainer* is a valid treatment of choice in mixed dentition where transverse expansion is a part of the treatment plan, as the results obtained are within short period of time and have less chances of relapse, because the correction of a malocclusion is by elimination of soft tissue dysfunction.

## 1. Introduction

The use of functional appliances has been reported since early times to produce skeletal and dentoalveolar changes [1–5]. These appliances are known to produce neuromuscular changes which lead to morphological modifications in the craniofacial complex [6–9]. A treatment based on only moving teeth is like treating only part of the problem and relapse could be expected. Thus, treatment plan intending to correct a malocclusion must include appliances which eliminate soft tissue dysfunction acting on the muscles of the cheeks, lips, and tongue, at the same time, correcting teeth and jaws position [10]. Furthermore, a two-phase orthodontic treatment, where a functional appliance is used to treat the functional problems and then brackets are used to align teeth, has been reported to improve relapse frequently occurring after orthodontic treatment with brackets [11]. During the last decades, better functional appliances have been developed and have been reported to produce significant changes in oral function as well as to stimulate mandibular growth [12]. The Trainer for Kids (T4K, Myofunctional Research Co, Australia) is a prefabricated functional appliance which is claimed to correct malocclusions at an early age by acting on muscular dysfunction and repositioning the mandible in forward direction and it stimulates the transverse development as well [13]. By initiating treatment at the mixed dentition stage, more treatment options are available and the need for complex orthodontic treatment involving permanent tooth extraction or orthognathic surgery is also minimized. They are also simple and economical. But the cases need to be carefully selected, and the operator should be well trained in their use.

Abundant information is available on preorthodontic trainer but less case reports have been reported in the literature that actually demonstrate the benefits of the trainer especially in mixed dentition. There is no literature published yet on the effect of trainer on end-on-molar relation in mixed dentition. The purpose of this paper is to present a clinical case where a patient with end on molar relation and proclination of teeth was successfully treated during the mixed dentition with preorthodontic trainer in a short period of time. Thus, this case report substantiates the importance of proper case selection and the use of preorthodontic trainer as a treatment modality in the correction of malocclusion in mixed dentition in children.

## 2. Case Report

An eleven-year-old female child of Asian origin was reported to the Department of Pedodontics, College of Dental Sciences, Davangere, Karnataka, India. The chief complaint of the patient was forwardly placed upper front teeth and irregular lower front teeth. The patient did not give any history of deleterious oral habits.

## 3. Diagnosis and Etiology

**Figure 1:**
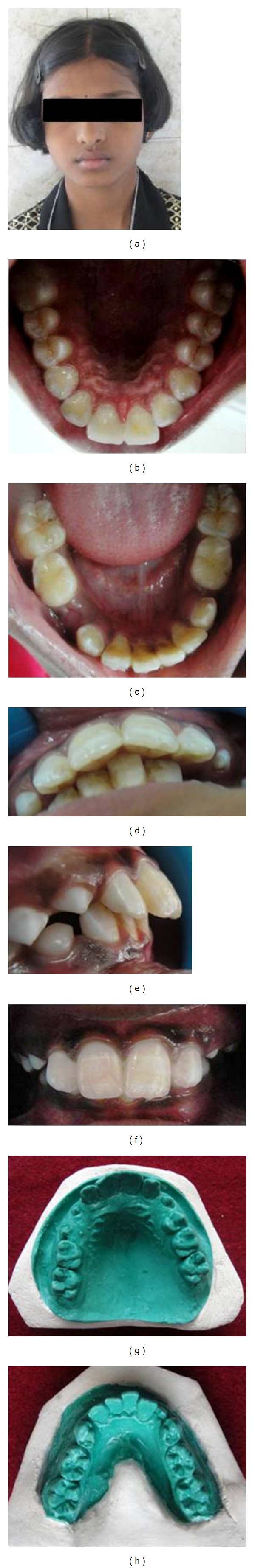
(a) Preoperative profile photograph. (b) Preoperative clinical photograph of maxillary arch. (c) Preoperative clinical photograph of mandibular arch. (d) Preoperative clinical photograph showing the upper and lower incisors in occlusion. (e) Preoperative clinical photograph showing the overjet. (f) Preoperative clinical photograph showing the overbite. (g) Preoperative study model of maxillary arch. (h) Preoperative study model of mandibular arch.

Extraoral examination revealed a convex profile, hyperactivity of the mentalis musculature, and potentially competent lips (Figure 1(a)). On intraoral examination, V-shaped upper arch (Figure 1(b)) and lower arch were seen (Figure 1(c)). Bilateral end on molar relation was present (Figures 3(a) and 3(b)). Proclination of the upper and lower teeth with 5.5 mm of overjet (Figure 1(e)) and overbite of 95% was present (Figure 1(f)). Crowding was seen in the lower anterior teeth and less space for the eruption of lower premolar and permanent canines (Figure 1(h)). Fluorosis stains were seen with anterior teeth. The mixed dentition analysis revealed that there was optimum space for the eruption of the maxillary premolars and for the mandibular arch there was deficiency of 2.5 mm of space. Steiner's cephalometric analysis was performed. Skeletal analysis showed values within normal range. Dental analysis showed increased upper incisor to NA (linear and angle), increased lower incisor to NB (linear and angle), and reduced interincisal angle (Figure 5(a)). There was no skeletal discrepancy; but dental analysis revealed proclination of upper and lower incisors. There were multiple problems to be corrected in this patient. Since the child was in mixed dentition, an appliance would redirect the growth as well as correct the existing problems.

## 4. Treatment Objectives

Treatment objective would include correcting the proclination of the maxillary teeth and relieving crowding in the mandible anterior teeth. Arch expansion was also desired to accommodate the erupting permanent teeth, correct the resting position of the tongue and improve the arch forms. Since dental and muscular problems were involved and the patient was in growing age, myofunctional appliance was planned for the patient. Preorthodontic trainer (Figure 6) was suggested to the patient as the child was in mixed dentition and there were no obvious skeletal discrepancies and she was found to be cooperative. The advantages, the amount of cooperation required, and the importance of regular use for the success of the treatment were explained to both the patient and the parents.

## 5. Treatment Progress

The patient was asked to wear the appliance for 1-2 hours in the day for two weeks and then overnight also. The patient was followed up for every 15 days for first two months and later once in every month.

## 6. Treatment Results

**Figure 2:**
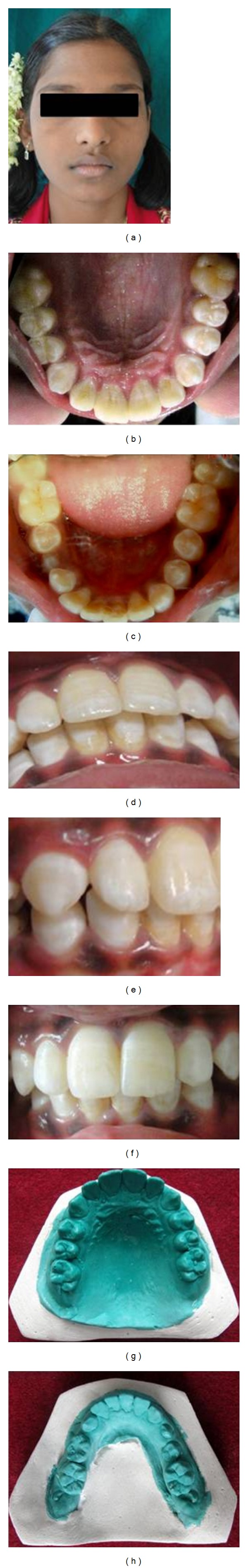
(a) Postoperative profile photograph. (b) Postoperative clinical photograph of maxillary arch. (c) Postoperative clinical photograph of mandibular arch. (d) Postoperative clinical photograph showing the upper and lower incisors in occlusion. (e) Postoperative clinical photograph showing the overjet. (f) Postoperative clinical photograph showing the overbite. (g) Postoperative study model of maxillary arch. (h) Postoperative study model of mandibular arch.

**Figure 3:**
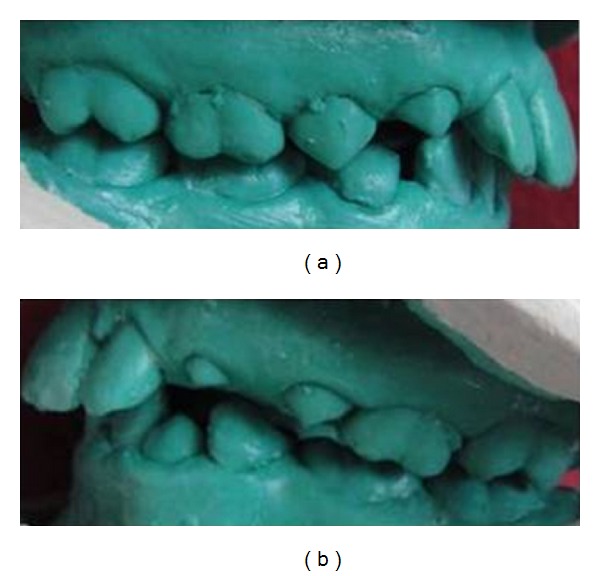
(a) Preoperative molar relation on the right side of the study model. (b) Preoperative molar relation on the left side of the study model.

**Figure 4:**
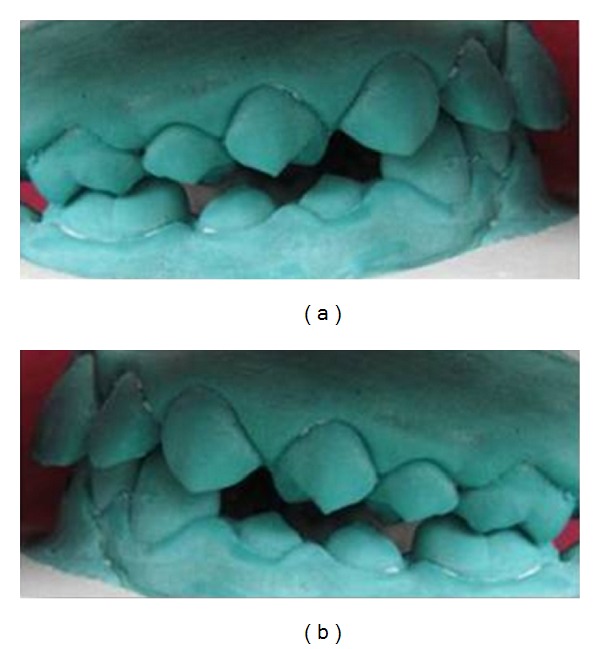
(a) Postoperative molar relation on the right side of the study model. (b) Postoperative molar relation on the left side of the study model.

**Figure 5:**
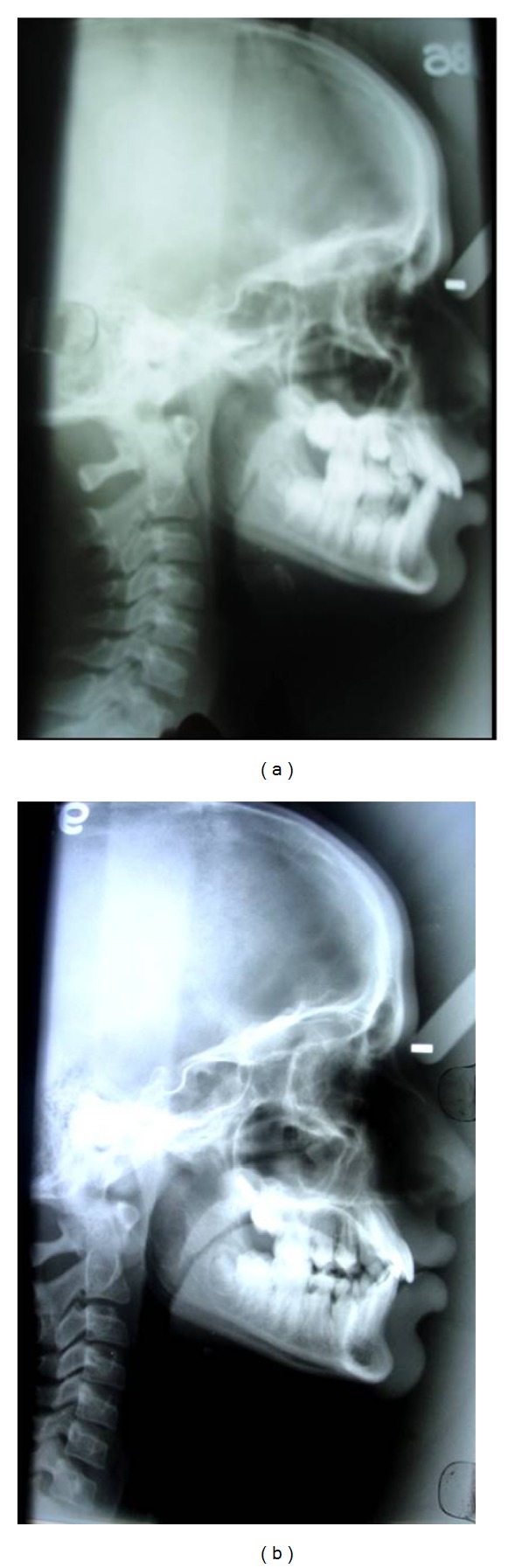
(a) Preoperative lateral cephalogram. (b) Postoperative lateral cephalogram.

**Figure 6:**
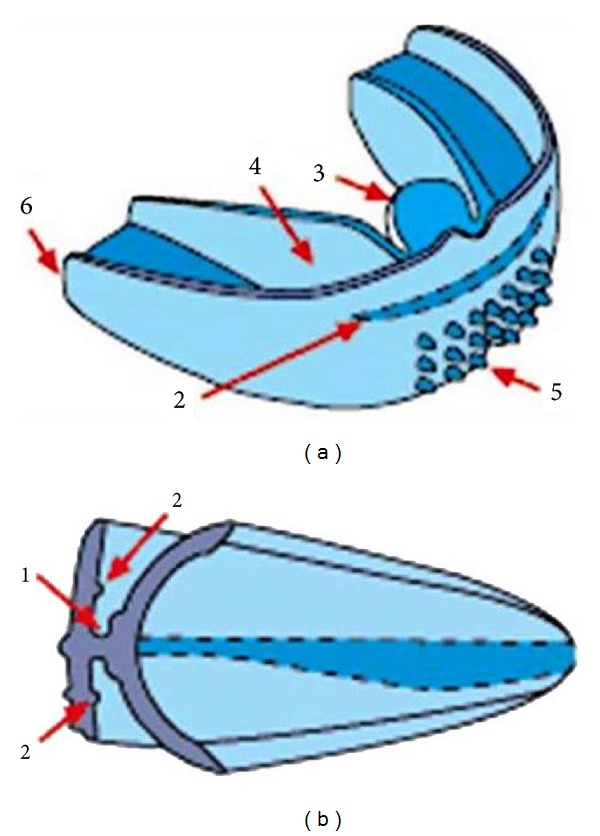
*Tooth guidance*: molded into the anterior section (similar to orthodontic archwire). 1—tooth channels. 2—labial bows (impart a light force on misaligned anterior teeth). *Myofunctional training*: 3—tongue tag (for the proprioceptive positioning of the tongue tip as in myofunctional and speech therapies). 4—tongue guard (stops tongue thrusting when being in place and forces child to breathe through the nose). 5—Lip bumpers (discourage overactive mentalis muscle activity). *Jaw positioning*: 6—edge-to-edge class I jaw position (is produced when in place (same as most functional appliances). Combined with prevention of tongue thrusting and forcing the child to nose breathe is how the class II corrections are achieved in the cases shown (a big assistance to the orthodontist in future treatment)).

Six months later, there were reduced mentalis activity and less convexity of the profile as the proclination of the teeth was reduced. The lips became competent (Figure 2(a)). There was an improvement in the upper and lower arches (Figures 2(b) and 2(c)). There was an increase in the intercanine distance which was evident by accommodating the erupting canine in the arch (Figures 2(g) and 2(h)). Crowding was relieved in the mandibular anterior teeth (Figure 2(h)). The molar relation changed from end on molar relation to Angle's class I molar relation (Figures 4(a) and 4(b)). The overjet was reduced from 5.5 mm to 2 mm (Figures 2(d) and 2(e)). There was reduction in the deep bite (Figure 2(f)). The interincisal angle increase was positively modified (Figure 5(b)). All these changes were seen just in a span of 6 months. The patient was asked to wear the same appliance for two more months as retention appliance. The patient was happy with the results of the treatment and did not opt for any fixed appliance therapy which was suggested to her for further fine detailing of the positions of the teeth. The parents of the patient also denied the treatment of the fluorosis stains that was suggested to them.

## 7. Discussion

Since there were no skeletal discrepancies, trainer was selected as a treatment of choice. Case selection is an important criterion for the success of the treatment with this appliance. The case presented here required the retraction of the anterior teeth, correction of the molar relation to Angle's class I, decrowding of the lower anterior teeth, transverse expansion of the maxillary and the mandibular arches, correction of muscular imbalances, and reduction of deep bite. According to model analysis, there was no space deficiency in the maxillary arch, but the retraction of the anterior teeth would require space and the decrowding of the lower teeth would also require space. The patient was in late mixed dentition with no skeletal discrepancies. All these corrections were expected out of use of single appliance. The trainer is often not able to offset major jaw discrepancies such as those at 11 years of age but the other dental effects of trainer were still desirable. The parents and the patient were cooperative and followed the instructions correctly. Hence the patient was put on preorthodontic trainer.

The clinical case presented showed that the preorthodontic trainer successfully treated an end on molar relation along with the proclination of the anterior teeth and deep bite in less than a year. The postoperative study models demonstrated well-aligned upper and lower arches (Figures 2(g) and 2(h)). The cephalometry demonstrated a positive modification in upper incisor to NA (linear and angle), lower incisor to NB (linear and angle), and interincisal angles within 6 months of treatment. The soft tissue dysfunction was eliminated and resting position of the tongue was also corrected. The treatment goals were achieved within half a year and further treatment became unnecessary.

The three effects of the trainer are tooth guidance, myofunctional training, and jaw positioning. It aligns the teeth and provides functional advantage. *Tooth guidance *in a trainer is made from a nonthermoplastic silicone or polyurethane which has both flexibility and inherent memory. The premoulded upper and lower labial bows have a similar effect to that of orthodontic arch wire. That is, they are premoulded to the parabolic shape of the natural arches and they adapt to any arch size, large or small. The labial bows combined with anterior tooth channels afford a constant force on misaligned anterior teeth to assist in the correction of their position. These components of the trainer might have caused the alignment of the teeth and hence the improvement in the arch forms in the clinical case presented here.

*Myofunctional training *effects of the trainer are correcting the incorrect tongue position and function, tongue thrusting, and oral habits which are the cause of many malocclusions. The design incorporates a tongue tag for proprioceptive location of the tongue tip. The raised section on the tag trains the child to place the tongue tip in the correct position with the trainer in place. This also acts as a “reminder” to place the tongue tip correctly without the trainer. The tongue guard prevents a tongue thrust swallow when being in place, which is a “position training” process for the tongue. Correction of these soft tissue problems has been shown to greatly influence growth, development, and long-term stability [10]. Lip bumpers or mentalis stretchers are incorporated to stretch and deactivate overactive mentalis contraction. Lip bumpers have been shown to gain arch length in mild to moderately crowded cases. This component of the trainer might have helped relieve the crowding in the lower arch in the present clinical case. The soft tissue problems were also taken care of by the tongue tag, tongue guard, and lip bumpers in the appliance.

The *jaw positioning *is by producing maxillary expansion which is accompanied by a spontaneous mandibular response, which increases the dental arch perimeter [14, 15] and rotates the mandible posteriorly [16–18]. The edge-to-edge class I jaw positioning of the appliance might have produced Angle's class I molar relation in the present clinical case.

In the present clinical case, the growth of the jaws might have also caused the desired effects but according to results of study by Ramirez and coworkers both maxillary and mandibular interpremolar and intermolar distances are significantly increased by the T4K, thus the arch perimeter providing more room for tooth alignment. It thus appears that the prefabricated functional appliance stimulates further transverse development overlapping that produced by natural growth, which may be an asset when treating patients with crowding caused by decreased maxillary or mandibular transverse development [19]. Thus, it may be inferred that most of the effects seen were because of the use of the preorthodontic trainer.

Another interesting finding in the present case is the reduction of the deep bite. Deep bite was diagnosed before the treatment which clinically got improved. This was a skeletal change and was confirmed with the McNamara analysis in which increase in the ANS-Me distance was observed (Figure 5(b)). This suggests that there was increase in the lower facial height. This result was mainly due to increase in maxillary arch dimensions which in turn caused mandibular growth. The mandible is relocated anteriorly and inferiorly by the remodeling at the condyle which clinically manifests as increase in the lower facial height and reduction in deep bite [13]. It can be inferred that skeletal changes can be a possibility in the late mixed dentition which was doubtful as mentioned in the earlier part of the discussion.

This paper only reports one patient. Controlled studies with an optimum sample have to be performed to confirm the actions of the T4K on various types of malocclusions. It is difficult to determine the reasons of the quick treatment success because the treatment is given in periods of accelerated growth [20]. But it can be inferred that the growth of the jaws as well as the position of the teeth can be guided to more favorable positions with the use of this appliance and the operator can be more certain of the results instead of waiting for the growth of the jaws to take place which may or may not take place.

## 8. Conclusion

It can be concluded that preorthodontic trainer permits treating several problems that are participating in the malocclusions development and thus permits treating the problem at different points. Case selection must be done with utmost care. The key to success of this appliance is *daily use *and there is no other substitute for the better results of this appliance.
